# Esophageal Achalasia: An Uncommon Complication during Pregnancy Treated Conservatively

**DOI:** 10.1155/2013/639698

**Published:** 2013-01-10

**Authors:** Dimitrios Spiliopoulos, Michail Spiliopoulos, Alero Awala

**Affiliations:** ^1^Obstetrics and Gynaecology Department, Watford General Hospital, 35 The Spinney, Watford, Hertfordshire WD17 4QF, UK; ^2^Department of Obstetrics, Gynecology and Reproductive Sciences, Temple University Hospital, 3401 N. Broad Street, Philadelphia, PA 19140, USA

## Abstract

A 38-year-old Caucasian woman, gravida 3 para 2, was admitted at 29 weeks of gestation because of vomiting, dysphagia for solids and liquids, and loss of weight. An enlargement of the anterior left neck region was noted on the palpation of the thyroid gland. An MRI of the neck showed a marked esophageal dilatation with the presence of food remnants along its length and the displacement of the trachea to the right. The findings of the upper gastrointestinal endoscopy and manometry were suggestive of esophageal achalasia. Conservative management with total parenteral nutrition (TPN) through a peripheral line proved to be successful. A healthy male baby was born by a cesarean section at 37 weeks. The patient underwent laparoscopic esophageal myotomy and fundoplication seven days postpartum.

## 1. Introduction 

Achalasia is a rare esophageal motility disorder involving the smooth muscle layer of the esophagus and the lower esophageal sphincter (LES). It is characterized by incomplete LES relaxation, increased LES tone, and nonperistaltic contractions of the esophageal body. The main symptoms are dysphagia, regurgitation, and vomiting. Chest pain, coughing, and loss of weight can be encountered also.

Achalasia can occur at any age but it usually presents between the third and fifth decade of life with the same rate between men and women [1] and a prevalence of 8 per million population [2]. The onset is insidious and that could be the reason why it rarely coexists with pregnancy. In pregnant women, it has been associated with maternal malnutrition and death, as well as preterm delivery, fetal growth restriction, and fetal demise [3].

Due to the rarity of achalasia in pregnancy, there are no data regarding perinatal management. We present a case of esophageal achalasia in the last trimester of pregnancy, managed with success with total parenteral nutrition (TPN) administered through a peripheral line.

## 2. Case Presentation 

A 38-year-old Caucasian woman, gravida 3 para 2, presented to our hospital with an intrauterine pregnancy at 29 weeks because of occasional vomiting, dysphagia for liquids and solids, anorexia, mild uterine contractions, and a nine kg weight loss over a 4-week period. Her first two children were delivered vaginally at term. Initial physical examination on admission revealed a fatigued woman with a weight of 62 kg, height of 178 cm, blood pressure of 110/75 mmHg, pulse 80 beats per minute, and temperature of 36.5°C. An enlargement of the left neck region was noted on palpation of the thyroid gland, with no enlarged peripheral lymph nodes. The rest of the physical examination was unremarkable. All laboratory investigations, including full blood count, liver function tests, and thyroid function tests were within normal range. Urine test results were unremarkable. 

The patient was admitted on the antenatal ward for observation and administration of i.v. fluids, antiemetics, and vitamins. A viable singleton pregnancy was confirmed by transabdominal ultrasonography with umbilical and uterine artery Doppler ultrasound velocimetry within normal limits. An estimated fetal weight (EFW) of 1681 gr was calculated via ultrasonography. 

**Figure 1:**
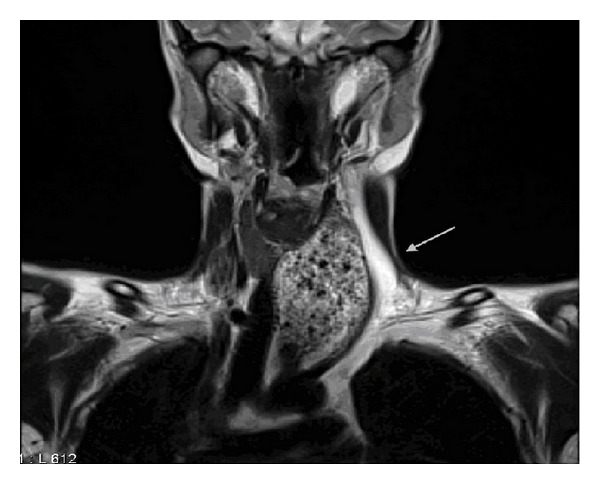
MRI scan of the neck region demonstrating a left palpable neck mass (arrow) with the presence of food remnants in the esophagus and displacement of the trachea to the right (coronal view).

**Figure 2:**
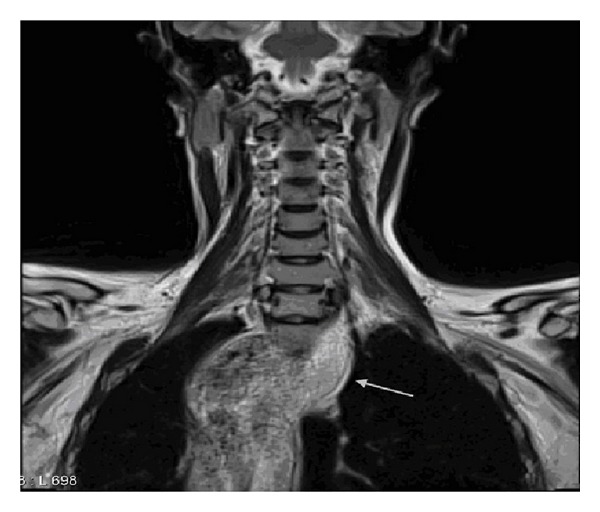
MRI scan of the neck region showing the extent of the esophageal dilatation (arrow) (coronal view).

Imaging studies included an ultrasound of the thyroid gland showing a normal-sized gland with the presence of a mass displacing the left thyroid lobe and the vessels of the left neck region. An MRI of the neck revealed a normal sized thyroid, larynx and lymph nodes, and a marked esophageal dilatation with the presence of food remnants along its length with displacement of the trachea to the right (Figures 1 and 2). 

The patient's symptoms did not improve over the next two days and a gastroenterologist was consulted. An upper gastrointestinal (GI) endoscopy showed an enlarged tortuous esophageal lumen, incomplete LES relaxation, esophagitis with difficult passage of the endoscope into the stomach, and no evidence of intraluminal esophageal compression. Examination of the second part of the duodenum was unremarkable. Esophageal manometry was also performed, showing an increased LES pressure of 44 mmHg with incomplete relaxation, consistent with achalasia. 

Treatment options to improve the nutritional status of both mother and fetus were discussed with the patient, including calcium channel antagonists, nitrates, pneumatic dilation, botulinum toxin injection, and parenteral nutrition (TPN). The woman denied any intervention during pregnancy and opted for TPN through a peripheral line of 2000 mL/day, with 1215 Kcal/day. During hospitalization, monitoring of the fetal condition was performed with cardiotocography and biophysical profile twice weekly. Fetal growth was within normal range and maternal body weight increased by two kg over the next eight weeks. An active, 2160 g male baby was delivered by a cesarean section at 37 weeks of gestation, with an Apgar score of 9. The baby was not admitted to neonatal intensive care unit and showed normal growing rate. The patient underwent laparoscopic myotomy and fundoplication seven days postpartum. Postoperatively, symptomatology from the digestive tract improved significantly and her appetite increased. She gained five kg of body weight in the next month. 

## 3. Discussion 

In pregnancy, the symptomatology of achalasia can be aggravated due to the physiologic changes in the LES and easily confused with gastroesophageal reflux or gravidarum. The diagnosis can be delayed considerably and the resulting maternal malnutrition can lead to adverse perinatal outcomes, such as fetal growth restriction, preterm delivery, and even fetal death [3]. Maternal mortality has also been reported [4]. Due to the rarity of this disorder, specific guidelines of management and treatment in pregnancy do not exist [2]. In addition, the age of onset of achalasia is varied and the time interval between the onset and clinical symptomatology can be unpredictable (from one to five years) [5]. 

The management options depend on the gestational age and severity of symptomatology. These include diet modification and medical therapy (calcium channel blockers and nitrates) in cases of late onset or mild disease, but they should be used with caution. The severity of achalasia in our patient precluded the use of medical therapy. Pneumatic dilation and injection of botulinum toxin in the LES are considered more effective measures until the delivery of the baby [6, 7]. However, they do pose some risks, such as esophageal rupture (3% risk) [8] and miscarriage after the use of botulinum toxin (category c medication) [9]. Wataganara et al. [10] have described the use of intersphincteric injection of botulinum toxin with the delivery of a healthy baby at 37 weeks by cesarean section. Various authors have reported cases of successful treatment of esophageal achalasia with endoscopic pneumatic balloon dilation with good perinatal outcomes [7, 11–13]. 

Cardiomyotomy (open or endoscopic) is another option but it carries the risk of general anesthesia and surgery and is contraindicated in pregnancy. It can be offered to the patient postpartum as a definite treatment. Palanivelu et al. [14] described the successful management of achalasia with laparoscopic Heller's myotomy during the second trimester of pregnancy. Ohno et al. [3] concluded that surgical myotomy can improve pregnancy outcome in women previously suffering from esophageal achalasia. Finally, Roca et al. [15] reported the use of an esophageal self-expanding prosthesis in a 36-year-old woman at 26 weeks gestation with good outcome (Table 1). 

 In our patient, where severe maternal malnutrition was noted, TPN can be used as a source of caloric source of glucose, lipids, electrolytes, and trace elements [16, 17]. In the past, TPN was thought to be the cause of uterine contractions and preterm labour [18]. Recent data suggest that increased preterm delivery rates observed in the past were a reflection of the underlying maternal condition, rather than direct consequence of the lipid component formulations for TPN [16]. Despite the risk of infection and thrombosis, maternal and neonatal outcome are not compromised by the use of TPN [19, 20]. Russo-Steiglitz et al. [18] had studied the nutritional status and maternal/neonatal outcome in ten patients with severe hyperemesis treated with TPN. They did not notice an increased incidence of intrauterine growth restriction (IUGR), preterm labor, or placental insufficiency in any of the patients studied. Additionally, recent progress in nutritional supplementation has shown that it can be used to defer definitive treatment after delivery [21]. Our case is supporting the importance of conservative management of esophageal achalasia during pregnancy with TPN, when definite surgical treatment cannot be applied. 

## 4. Conclusion 

Achalasia in pregnancy remains a rarely encountered condition. Esophageal achalasia should be suspected in pregnant women presenting with dysphagia for solids and liquids, occasional vomiting, and loss of weight. The management and treatment needs to be individualized and its advantages and disadvantages discussed thoroughly with the patient. Total parenteral nutrition in pregnancy is a safe option of conservative management of achalasia and it has not been associated with adverse perinatal outcomes such as preterm delivery, growth restriction, or increased perinatal mortality. It remains a valid and effective choice of management to any other intervention during pregnancy, especially when the patient does not opt for a surgical treatment.

## Figures and Tables

**Table 1:** Reported cases of achalasia in pregnancy with the type of treatment and pregnancy outcome (from 1969 till 2010).

Author	Year	Number of cases	Age of pt/weeks gestation	Type of treatment	Outcome
Paulsen et al.	2010	1	34 yo/?	Balloon dilation	Uncomplicated birth

Wataganara et al.	2009	1	39 yo/33 w	Botulinum toxin	35 w C-section

Diaz Roca et al.	2009	1	36 yo/26 w	Self expanding prosthesis	Uneventful delivery

Palavinelu et al.	2008	1	24 yo/2nd trimester	Laparoscopic Heller's myotomy	Healthy baby

Pulanic et al.	2008	1	30 yo/26 w	Pneumatic dilation	Vaginal delivery 38 w

Ohno et al.	2000	1	34 yo/27 w	Surgical myotomy postpartum	Intrauterine fetal death

Kalish et al.	1999	1	42 yo/31 w	Antifungal medication	Spontaneous vaginal delivery 38 w

Fassina et al.	1995	1	23 yo/24 w		Unexplained sudden maternal death (6-month pregnancy), megaesophagus

Fiest et al.	1993	1	24 yo/8 w	Balloon dilation	Spontaneous vaginal delivery 35 w (healthy infant)

Satin et al.	1992	1	28 yo/38 w	Pneumatic dilation	Induced vaginal delivery 38 w (healthy infant)

Mayberry and Atkinson	1987	20	18 yo–45 yo (mean 32 yo)/?	Comparison of reproductive histories of women with achalasia with those of a control group	No significant difference in the number of live births of patients versus controls 3 miscarriages after diagnosis

Clemendor et al.	1969	10	22 yo–37 yo (mean 29.1 yo)/24 w–30 w	1case Pneumatic dilation (1st report) 5 cases Bougie dilation 3 cases medical treatment 1 case no treatment	Living offspring in only 5 cases (the rest: 2 terminations, 2 stillbirths, 1 premature delivery).
